# Monitoring quality of care for peripheral intravenous catheters; feasibility and reliability of the peripheral intravenous catheters mini questionnaire (*PIVC-miniQ*)

**DOI:** 10.1186/s12913-019-4497-z

**Published:** 2019-09-05

**Authors:** Lise Husby Høvik, Kari Hanne Gjeilo, Stian Lydersen, Claire M. Rickard, Benedikte Røtvold, Jan Kristian Damås, Erik Solligård, Lise Tuset Gustad

**Affiliations:** 1 Department of Circulation and Medical Imaging, Norwegian University of Science and Technology (NTNU), Postbox 8905, 7491 Trondheim, Norway; 2 Gemini Center for Sepsis Research, St. Olavs Hospital and Norwegian University of Science and Technology (NTNU), Trondheim, Norway; 3 Clinic of Anaesthesia and Intensive Care, St. Olavs Hospital, Trondheim, Norway; 4 Department of Cardiothoracic Surgery, Department of Cardiology and National Competence Centre for Complex Symptom Disorders, St. Olavs Hospital, Trondheim University Hospital, Trondheim, Norway; 5 Department of Public Health and Nursing, Faculty of Medicine and Health Sciences, Norwegian University of Science and Technology (NTNU), Trondheim, Norway; 6 Regional Centre for Child and Youth Mental Health and Child Welfare, Department of Mental Health, Faculty of Medicine and Health Sciences, Norwegian University of Science and Technology (NTNU), Trondheim, Norway; 7 Alliance for Vascular Access teaching and Research, Menzies Health Institute Queensland, Griffith University, Brisbane, Australia; 8 School of Nursing and Midwifery, Griffith University, Brisbane, Australia; 9 Department of Anesthesia, Levanger Hospital, Clinic of Surgery, Nord-Trøndelag Hospital Trust, Levanger, Norway; 10 Centre of Molecular Inflammation Research, Department of Clinical and Molecular Medicine, NTNU, Trondheim, Norway; 11 Department of Infectious Diseases, St. Olavs Hospital, Trondheim, Norway; 12 Department of Medicine, Levanger Hospital, Clinic of Medicine and rehabilitation, Nord-Trøndelag Hospital Trust, Levanger, Norway

**Keywords:** Peripheral intravenous catheter, Quality assessment, Quality improvement

## Abstract

**Background:**

Peripheral intravenous catheters (PIVCs) account for a mean of 38% of catheter associated bloodstream infections (CABSI) with *Staphylococcus aureus*, which are preventable if deficiencies in best practice are addressed. There exists no feasible and reliable quality surveillance tool assessing all important areas related to PIVC quality. Thus, we aimed to develop and test feasibility and reliability for an efficient quality assessment tool of overall PIVC quality.

**Methods:**

The Peripheral Intravenous Catheter- mini Questionnaire*, PIVC-miniQ,* consists of 16 items calculated as a sum score of problems regarding the insertion site, condition of dressing and equipment, documentation, and indication for use. In addition, it contains background variables like PIVC site, size and insertion environment. Two hospitals tested the *PIVC-miniQ* for feasibility and inter-rater agreement. Each PIVC was assessed twice, 2–5 min apart by two independent raters. We calculated the intraclass correlation coefficient (ICC) for each hospital and overall. For each of the 16 items, we calculated negative agreement, positive agreement, absolute agreement, and Scott’s pi.

**Results:**

Sixty-three raters evaluated 205 PIVCs in 177 patients, each PIVC was assessed twice by independent raters, in total 410 PIVC observations. ICC between raters was 0.678 for hospital A, 0.577 for hospital B, and 0.604 for the pooled data. Mean time for the bedside assessment of each PIVC was 1.40 (SD 0.0007) minutes. The most frequent insertion site symptom was “pain and tenderness” (14.4%), whereas the most prevalent overall problem was lack of documentation of the PIVC (26.8%). Up to 50% of PIVCs were placed near joints (wrist or antecubital fossae) or were inserted under suboptimal conditions, i.e. emergency department or ambulance.

**Conclusions:**

Our study highlights the need for PIVC quality surveillance on ward and hospital level and reports the PIVC-miniQ to be a reliable and time efficient tool suitable for frequent point-prevalence audits.

**Electronic supplementary material:**

The online version of this article (10.1186/s12913-019-4497-z) contains supplementary material, which is available to authorized users.

## Background

Peripheral intravenous catheters (PIVCs) are the most used intravascular devices in hospitals, as up to 80% of hospitalized patients require intravenous (IV) therapy [1]. When managed properly, PIVCs are safe devices with little risk for serious complications. However, PIVC complications such as phlebitis, extravasation, infiltration and infections are common [2] and infected PIVCs account for a mean of 38% of catheter associated blood stream infections (CABSI) caused by *Staphylococcus aureus* (*S. aureus*) [1]. Preventable CABSI and sepsis are serious complications with high mortality [3–6], estimated to occur in 0.5 cases per 1.000 PIVC catheter days [7]. Considering the high proportion of PIVCs in clinical practice, even a low incidence of CABSI can still give a considerable impact [4, 8–12].

A large international multicenter study, The One Million Global catheters study (OMG), revealed poor current practices regarding insertion and management of PIVCs [13, 14]. In the OMG study, PIVCs were in use despite signs of local infection and pain, a large amount of PIVCs were kept without indication, and securement dressings were often blood stained or loose. Documentation of these complications were often lacking in patient journals [13, 14]. All these factors have been shown to increase infection risk and hamper patient safety and are not compliant with best practice [15].

Today, many screening tools are available for PIVC phlebitis assessment, and at least 71 phlebitis scales exists [16–18]. Recently, large studies have measured complications and risk factors regarding PIVC care [19, 20]. However, there exist no feasible and reliable quality surveillance tool assessing all important aspects of PIVC quality; including signs of local infection and pain, information on redundant catheters, lack of documentation of PIVC insertion date or poor condition of securement dressing and equipment connected to the PIVC [18, 21, 22]. A simple quality surveillance tool, reliable across raters, mirroring deviation in overall PIVC quality, can be used by ward or hospital managers in order to raise front line personnel’s risk awareness of these issues.

Health care organizations are challenged in creating systems for constant improvement [23]. In the present study, we used questions from comprehensive point-prevalence questionnaires [13, 21] in order to develop a quality assessment tool suitable for repeated measures aiming at reporting rates and magnitude of undesired PIVC practice. Further, the tool’s objective is to measure the effect of interventions, targeted at improving overall PIVC quality at the ward or hospital level. We labeled the tool Peripheral Intravenous Catheter mini questionnaire *(PIVC-miniQ*) (Additional file 1: Figure S1). The *PIVC-miniQ* is designed for repeated point-prevalence audits to improve and monitor quality. The overall goal of this study was to assess the feasibility and inter-rater agreement of the *PIVC-miniQ* in clinical practice.

## Methods

In this study, feasibility is defined as time to complete the *PIVC miniQ* and number of missing values on each item [18]. Reliability is defined as inter-rater reliability i.e. the instruments ability to produce agreement between two raters that assess the same PIVC blinded from each other’s ratings [24].

### Study sites

Two hospitals in Mid-Norway participated. Hospital A is the local hospital for 100,000 inhabitants and hospital B is the local hospital for 280,000 and referral hospital for 700,000 inhabitants. Both hospitals share the same documentation systems. Even though the two hospitals have electronic patient journals, observations regarding PIVCs and medication are on paper-based observation curves that are scanned into the patient record after the hospital stay is over.

In 2015, Hospital A used front-line, research and education nurses to develop a short assessment tool for PIVC quality surveillance, based on experience from their participation in the OMG study [14]. The main target was local quality surveillance. Hospital A used the pre-version of the *PIVC-miniQ* in three point-prevalence audits. The poor baseline quality was presented at safety huddles at each ward using Epidata time series to display the sum score for each PIVC and mean of problems across all PIVCs. These time series were also used in a 30-min in-house training session about the gap between desired and observed PIVC quality. On the second and third *PIVC-miniQ* audit, one and two months after these improvements` actions took place, the measurements showed improved quality of PIVC care (Additional file 1: Figure S2).

As the *PIVC-miniQ* pre-version showed promise to enable overall PIVC quality improvements, we wanted to develop and test feasibility and reliability for the tool in a major context, and in 2017, a university hospital (B) was included.

### PIVC-miniQ

The *PIVC-miniQ* underwent a revision, based on Hospital A’s experience, search for literature and guidelines regarding items mirroring PIVC quality [13, 16, 18, 21], together with discussions with IV team and expert nurses in Hospital B. The selection of the final items was based on thorough consideration and consensus in the group. For detailed overview of the development process, see Additional file 1: Table S1.

The final version of the *PIVC-miniQ* consists of four main assessment areas (Additional file 1: Figure S1). First area reflects phlebitis-related signs and symptoms; insertion site (9 items; including pain or tenderness, redness, swelling, warmth, purulence and hardness of tissue) [25, 26], where signs are assessed by the rater (redness, swelling etc.) and symptoms are expressed by the patient (pain and tenderness). Second area reflects PIVC dressing and IV connection related to PIVC failure [26] (5 items; soiled dressing, dressing with loose or lifting edges, blood in line and absence of insertion date on PIVC dressing). Further, it provides two assessment areas regarding process of care: lack of documentation of PIVC insertion in the patient journal (1 item), and if there is indication for use defined as procedures requiring a PIVC, even if it is not currently used; for instance when the patient has epidural for pain treatment or is connected with telemetry due to irregular heart function (1 item). This makes the *PIVC-miniQ* a hybrid of dimensions of processes (clinical practice and ability to follow procedures) and outcomes (PIVC related signs and symptoms). Each problem that is present accounts for 1 point, and all problems are summarized in an overall score (scale range 0–16) that can be used for point prevalence audits of overall PIVC quality. An overall score of 0 indicates very good PIVC quality and should be the goal for clinical practice.

In addition, two dimensions of process in clinical practice were added as background variables due to clinical evidence; PIVC placement and PIVC insertion environment. PIVC insertion is not recommended near joints [27] and PIVC inserted in emergency settings are advised to be replaced [1]. We find these variables important on a system level as high prevalence of these factors in a hospital may communicate a questionable practice. These variables are not part of the *PIVC-miniQ* total score but are useful both in research and prevalence audits and can be analyzed separately for identifying items of concern.

According the aim for inter-rater agreement, we made it mandatory to mark “yes” or “no” for each PIVC statement (either the problem exists or not) since proportions of positive and negative agreement between raters are important for evaluating an assessment instrument.

### Selection of patients and raters

We collected PIVC characteristics from medical and surgical patients able to provide verbal consent across two hospitals. A few isolated infectious patients were excluded as isolation procedures are time consuming and we wanted to measure the time spent on the questionnaire. Data were collected between September 2017 and March 2018 from a convenience sample of patients for PIVC site assessment. Every available patient with PIVC on the wards, except the former mentioned were assessed, which makes it a population-based sampling.

For the inter-rater testing we used nurse educators, bedside nurses or nurse students, and mixed the raters as far as possible. None of the raters had been involved in the insertion of the PIVC they assessed. This heterogeneous group was chosen by purpose as we wanted to realistically assess agreement between potential users of the *PIVC-miniQ*. There was no training of personnel prior to data collection, only a brief explanation of the instrument. Hospital A had however some experience from their use of the pre-version of the *PIVC-miniQ*. Hospital B had sparse experience with PIVC assessment but was participating in further revisions of the *PIVC-miniQ*.

### Testing procedure

Each PIVC was assessed twice; 2–5 min apart between two independent raters in random sequencing, i.e. the raters mixed between being first and second to assess the PIVC. The first rater explained the procedure to the patient which also was provided a pamphlet of information of the study, and then asked for verbal consent. Thereafter, the assessment was performed bedside while observing the PIVC site. The second rater was meanwhile assessing another patients PIVC as first rater. Both raters collected background variables to be sure the same PIVC was assessed and recorded. The time between assessments was held as short as possible to make sure the PIVC was in the same state at both assessments and to ensure that the patient was not discharged or was away to some procedure or diagnostics. After observing the PIVCs bedside, the paper-based record was considered for documentation of the PIVC or if the patient had any indication for the PIVC that were not obvious bedside. The data was collected on paper and the raters instructed not to discuss or compare their ratings, and imminently after sampling put data sheet in a folder in a locked closet, waiting to be plotted in SPSS.

### Statistical analyses

Descriptive statistics regarding the raters, patients, PIVCs, score on each item and time to complete the *PIVC miniQ* are reported as frequencies (n) and proportions (%) for categorical data and mean (SD) for continuous data. We used ANOVA test for subgroups of raters. The distribution of the sum score for all PIVCs is shown in a histogram. Feasibility is presented as the number of missing data and time to complete the *PIVC-miniQ*. Missing values on single items were singly imputed using the Expectation maximation (EM) algorithm, using the 16 items on the *PIVC-miniQ* as predictors. Imputed values were thereafter rounded up to nearest integer 0 (problem does not exist) or 1 (problem exist). PIVCs located near joints, i.e. in antecubital fossa or wrist, was defined as an undesirable PIVC anatomic location, large PIVCs were defined as 14–18 G and PIVCs inserted under suboptimal conditions as prehospital or emergency departments were regarded objectionable on a system-level. For the sum scores, the degree of agreement was quantified as intraclass correlation coefficient (ICC). All ICC formulas consist of a ratio of variance [28]. We used a mixed model with PIVC sum score as the dependent variable, and PIVC and rater as crossed random effects. For ICC we used the correlation estimate provided by Rabe-Hesketh and Skrondal 2012, p. 437–441 [29], ICC (rater) = Variance component PIVC/ (Variance component PIVC + Variance component rater + residual variance). For each of the 16 items, we calculated negative agreement, positive agreement, absolute agreement, and Scott’s pi. Negative agreement can be interpreted as the probability that the second rater classifies “no” given that the first rater classifies the same as “no” [29]. Positive agreement is interpreted correspondingly. Absolute agreement is the probability that two raters evaluate the same item in the same category (yes/no). We also report Scott’s pi which is an agreement measure adjusted for chance [30]. We chose Scotts pi and not Cohen’s kappa for the latter purpose: As pointed out by DeVet [31], Cohens Kappa has some weaknesses regarding multiple raters and is also affected by bias [30, 32]. Statistical analyses were carried out using Stata 15.1 and SPSS 25.

## Results

A total of 177 patients across two hospitals and 17 wards had their PIVCs screened by two raters using the *PIVC-miniQ*. The wards consisted of units of orthopedic, gastroenterological, urological and thoracic surgery as well as general medical units. Twenty-five patients had 2 PIVCs and three patients had 3 PIVCs. This resulted in 205 PIVCs, i.e. a total of 410 PIVC observations. For the procedure we recruited 63 raters, from experienced nurse educators to nursing students. The number of observations per rater ranged from 1 to 128 PIVCs (median 17). For the heterogeneous group of raters, subgroup analyses and ANOVA test between groups of raters were non-significant (*p* = 0.289). There were missing data in 6.5% of the cases and in 0.4% of all items. The item with most missing was documentation, with 15 missing observations out of 410 (3.6%).

**Fig. 1:**
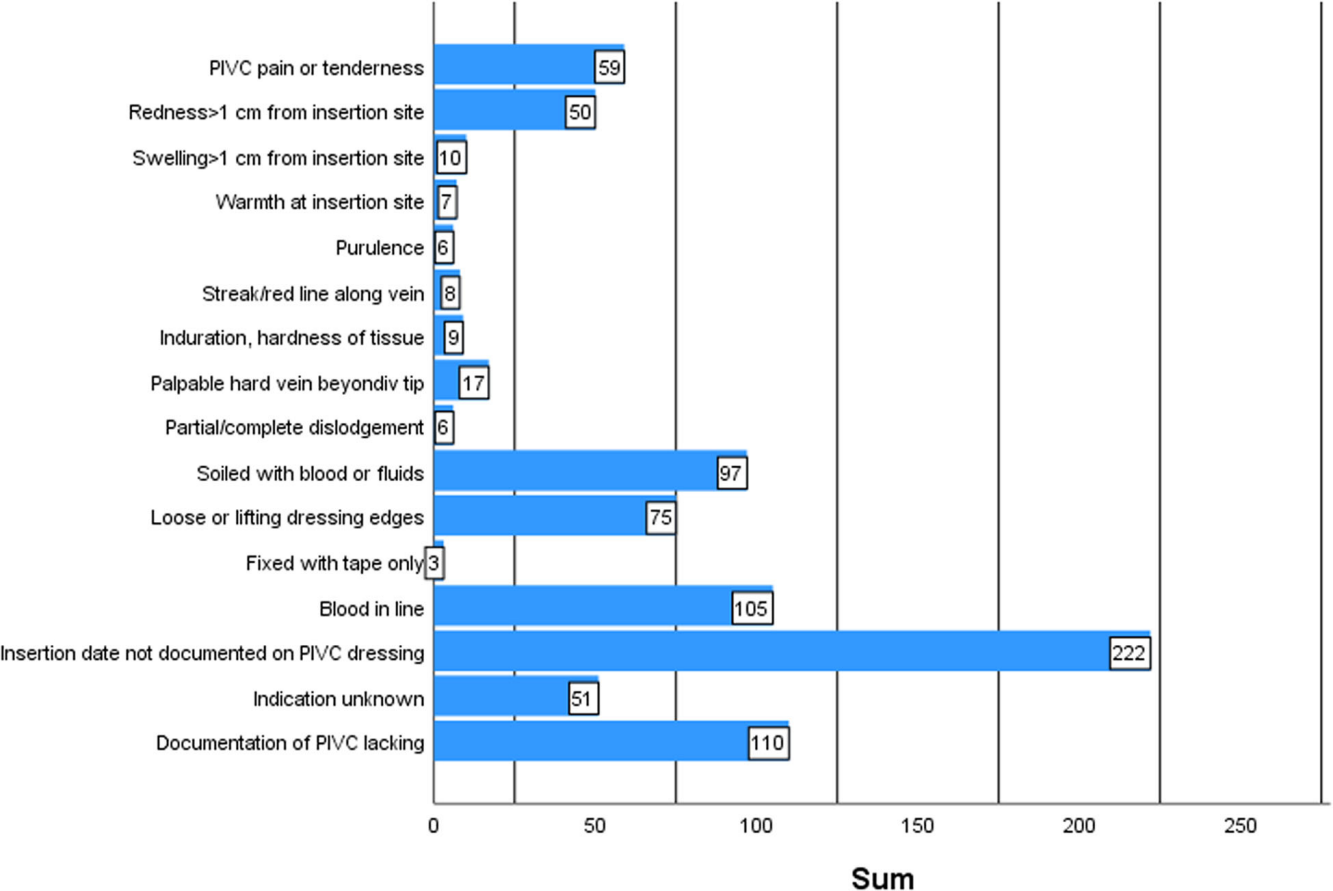
Frequency distributions for each of the 16 variables in the *PIVC-miniQ* (*n* = 410)

**Fig. 2:**
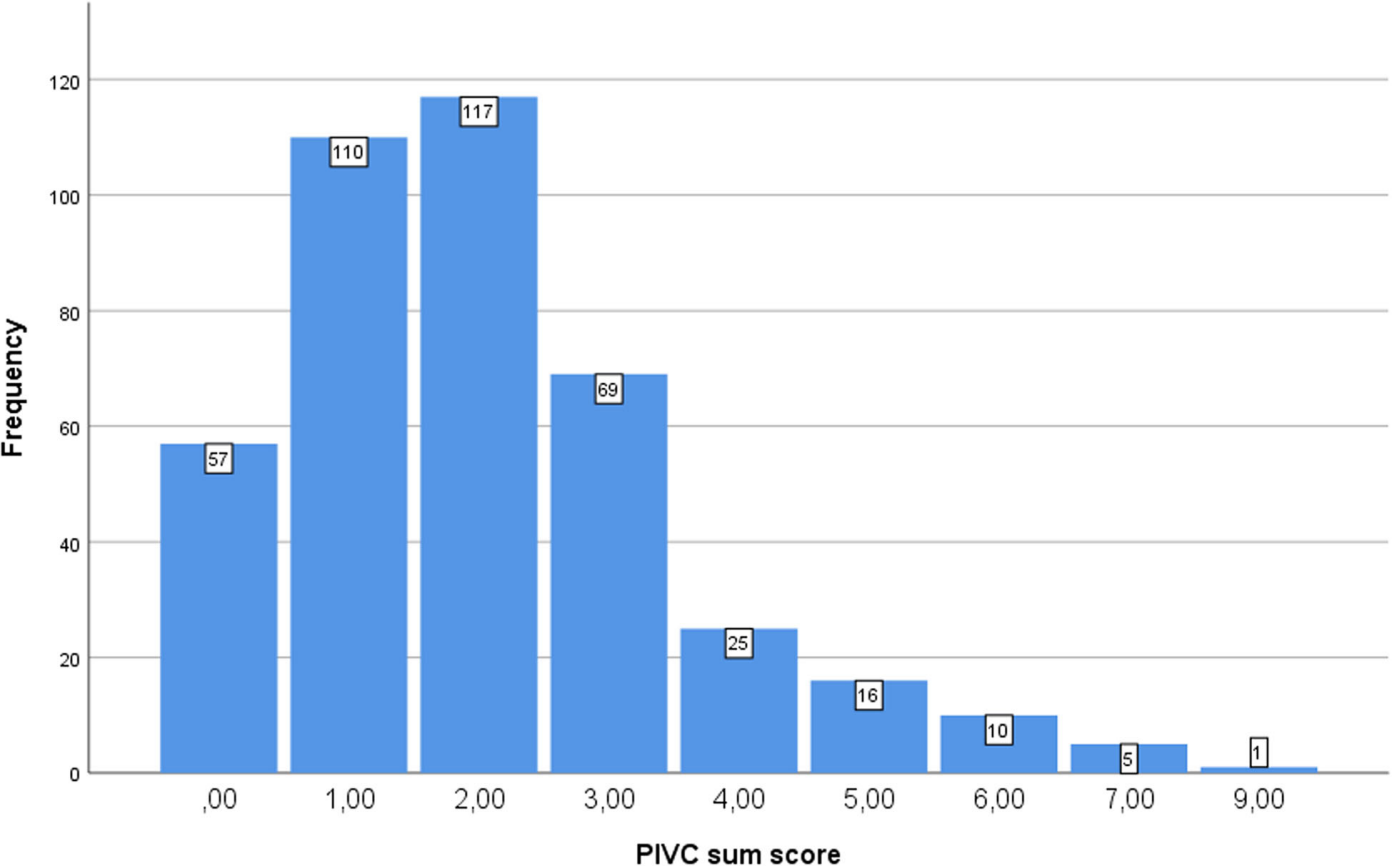
*PIVC-miniQ* sum score for all PIVCs assessed (n = 410)

Time used for the bedside assessment of the PIVC was 1.40 (SD 0.0007, minimum 30 s and maximum 5 min) and time used for finding the relevant information regarding the documentation item was 1.39 (SD 0.0009, minimum 15 s and maximum 6 min). Descriptive statistics for raters, patients and PIVCs are presented in Table 1. Observations of all the 410 PIVCs shows that in hospital B, 48.3% of PIVCs were inserted in undesirable anatomical locations near joints (wrist or antecubital fossa), and 46.8% of the PIVCs were larger than the recommended size, i.e. 20G or larger. In hospital A, 55.1% of the PIVCs remained in place despite insertion in objectionable environments. Indwell time of PIVCs varied from 0 to 9 days (median 1.0). The distributions for each of the 16 variables are shown in Fig. 1 (data from all 410 observations). The most frequent insertion site symptom was “pain and tenderness” (14.4%), next “redness” (12.2%), whereas the most prevalent overall problem was lack of documentation of the PIVC (26.8%). Figure 2 shows the distribution of the *PIVC-miniQ* sum score for all PIVCs assessed (*n* = 410) where a sum score of 9 was the worst measured, and overall mean score was 2.04 (SD 1.55). As shown in Table 2, the ICC between raters was 0.678 for hospital A and 0.577 for hospital B and 0.604 for the pooled data.

**Table 1:** Descriptive statistics* for raters, patients and PIVC background *n* = 410

	Hospital A	Hospital B	Total
Raters			
Number of raters	10	54	64
Nurse student	1 (10.0)	3 (5.6)	4 (6.3)
Primary nurse	3 (30.0)	46 (85.2)	49 (76.6)
Educator nurse	6 (60.0)	5 (9.3)	11 (17.1)
Patients			
Number of patients	32	145	177
Patient Age, mean (SD)	70.7 (15.4)	63.1 (17.1)	64.9 (16.9)
Patient Gender			
Female	22 (68.8)	66 (45.5)	88 (49.7)
Male	10 (31.2)	79 (54.5)	89 (50.3)
PIVC			
Number of PIVCs	49	156	205
Ward unit			
Medicine	27 (55.1)	56 (35.9)	83 (40.5)
Surgery	22 (44.9)	100 (64.1)	122 (59.5)
PIVC indwell time (days) mean (SD), range	1.50 (1.1) 0–9	1.95 (1.6) 0–7	1.90 (1.6) 0–9
PIVC site			
Hand	18 (36.7)	34 (21.8)	52 (25.4)
Wrist	13 (26.5)	21 (13.5)	34 (16.6)
Forearm	10 (20.4)	41 (26.3)	51 (24.9)
Antecubital fossa	8 (16.3)	57 (36.5)	65 (31.7)
Foot	0	2 (1.3)	2 (0.9)
Upper arm	0	1 (0.6)	1 (0.5)
PIVC size (gauge)			
22 G	9 (18.4)	31 (19.9)	40 (19.5)
20 G	28 (57.1)	41 (26.3)	69 (33.7)
18 G	11 (22.4)	82 (52.6)	93 (45.3)
16 G	1 (2.0)	2 (1.2)	3 (1.5)
Insertion Environment			
Ambulance/ EMS	6 (12.2)	8 (5.9)	14 (6.8)
Emergency department	21 (42.9)	34 (21.8)	55 (26.8)
Operating theatre	4 (8.2)	45 (28.8)	49 (23.9)
Hospital ward/unit/ICU	14 (28.6)	60 (38.5)	74 (36.1)
Radiology/ procedure room	1 (2.0)	1 (0.6)	2 (1,0)
Unknown	3 (6.1)	8 (5.1)	11 (5.4)

*N (%) shown unless otherwise indicated

Abbreviations: *PIVC* Peripheral Intravenous Catheter, *G* Gauge, *EMS* Emergency Medical Services

*ICU* Intensive Care Unit

**Table 2:** Variance components and the resulting ICC for the *PIVC-miniQ*

Variance component	Hospital A	Hospital B	All
PIVC	1.687	1.450	1.507
Rater	0.105	0.381	0.297
Residual	0.695	0.680	0.692
ICC (Rater)	0.678	0.576	0.604

Abbreviations; *PIVC* Peripheral Intravenous Catheter, *ICC* Intra Class Correlation

Specific positive and negative agreement and the agreement coefficient, Scott’s pi on each item are presented in Table 3. “Pain and tenderness” was the insertion site item with the highest level of positive agreement between raters, followed by redness. Other signs of local infection had poor positive agreement, and these also had a low prevalence of symptoms. There was however consistent negative specific agreement for each item (agreement on absence of symptoms). The dressing and documentation items had good overall positive and negative agreement.

**Table 3:** Agreement and reliability results for the 16 items of the Peripheral Intravenous Catheters mini questionnaire (PIVC miniQ)

Item on the PIVC miniQ	neg	disagree	positive	sum	Negative agreement	Positive agreement	Positive agreement	Scott’s agreement*
PIVC Pain and tenderness	168	15	22	205	0.957	0.746	0.927	0.754
Redness > 1 cm from insertion site	167	26	12	205	0.928	0.480	0.873	0.786
Swelling> 1 cm from insertion site	195	10	0	205	0.975	0.000	0.951	0.952
Warmth at insertion site	199	5	1	205	0.988	0.286	0.976	0.966
Purulence	200	4	1	205	0.990	0.333	0.981	0.971
Streak/red line along the vein	199	4	2	205	0.990	0.500	0.981	0.962
Induration, hardness of tissue	197	7	1	205	0.983	0.222	0.966	0.957
Palpable hard vein beyond tip	190	13	2	205	0.967	0.235	0.937	0.921
Partial/complete dislodgement	201	2	2	205	0.995	0.667	0.990	0.971
Soiled with blood or fluids	146	21	38	205	0.933	0.784	0.898	0.639
Loose or lifting dressing edges	156	23	26	205	0.931	0.693	0.889	0.701
Fixed with tape only	202	3	0	205	0.993	0.000	0.985	0.986
Blood in line	133	39	33	205	0.872	0.629	0.810	0.619
Insertion date not documented on PIVC dressing	85	18	102	205	0.904	0.919	0.912	0.503
Indication unknown	169	21	15	205	0.942	0.588	0.898	0.782
PIVC insertion date in is chart is lacking	132	36	37	205	0.880	0.673	0.824	0.607

* Bias adjusted Scott’s agreement

*PIVC* Peripheral Intravenous Catheter

## Discussion

We found that the *PIVC-miniQ* was feasible; it was quick with little missing data and a reliable and efficient process measure for quality control, taking only 1 min and 40 s on average bedside. The measure of consistency can be described as moderate to high with an ICC of 0.604 for the sum score. Total and negative specific agreement for each item was excellent, but there was inconsistency in positive specific agreement, especially on the items capturing problems with low positive prevalence. However, as the sum score was consistent across individual raters, the *PIVC- miniQ* can be used to reliably measure development in PIVC overall quality in point-prevalence audits and to be used to improve patient safety.

Observer variation is a challenge in clinical quality assessment. Goransson et al.`s study of measurement tools for phlebitis found that the proportion of PIVC phlebitis varied within and across instruments [17]. Furthermore, most phlebitis scales consists of grades of assessments, i.e. degree of redness or swelling which makes them questionable in clinical settings [17]. Even when using “yes” and “no” in our study, we found it surprising that items that seemed easy to objectively assess, like “blood in line” or “PIVC insertion date in chart is lacking” only had 0.63 and 0.67 in positive agreement, respectively.

Most studies have used a limited number of educated raters [16, 17, 21] but the absolute agreement has remained elusive [16, 17]. Our use of multiple raters mirrored the real-life assessment situation where individual staff education and experience in PIVC assessment varies widely. Using multiple nurses most likely resulted in a great discrepancy between the raters. Nevertheless, the overall *PIVC-miniQ* score in our study had good inter-rater reliability and the nurses did observe problems with the PIVCs, even if they categorized the problems differently.

By using the *PIVC-miniQ* in clinical practice, we were able to show that many PIVCs are kept in place despite pain and redness around insertion site, blood in the line, and dressings not changed despite being soiled with blood and fluids. This is disturbing given the short dwell (average 1.8 days), but similar results were identified in the OMG study, assessing PIVCs worldwide [14]. Our study also revealed a high prevalence of stable patients with PIVCs inserted in emergency settings, despite the recommendation to replace such PIVCs as early as possible due to the possibility of non-aseptic insertion [25, 33]. If the patient still is critically ill and there had been no time to replace it, such findings are inevitable but a high prevalence on a hospital level gives reason for concern. Additionally, a high proportion PIVCs were placed near joints and defined as large size; both considered as an undesirable practice linked to premature device failure [4, 9]. Finally, undocumented and redundant PIVCs were a problem, which seems to be a worldwide issue in need of improvement [14]. A high overall prevalence of PIVC problems are however a quality issue that must be addressed as a common responsibility for all health personnel. These findings are clinically relevant, describing patient important quality problems well suited for quality improvement projects that the leaders can use to motivate and educate health personnel towards change and best practice [22, 34]. Frequent feedback on quality of care may contribute to improved patient safety [35]. Audits discovering PIVC care of low quality must call for an action and be discussed in safety huddles or in-house training sessions and may contribute to improved quality over time [36], as shown in Hospital A.

Learning from malpractice in patient care is essential for improving patient safety. Using the *PIVC -miniQ* in feedback loops for frontline health personnel, can create a platform of learning that benefits the patients. Investing in PIVC quality improvement is important as healthcare associated CABSI is preventable when knowledge on best practice is addressed. Surveillance of the quality related to ubiquitous devices as PIVCs in healthcare are, therefore, of utmost importance [3, 4, 9, 37–40]. Each item of the *PIVC-miniQ* gives indications of recommended practice, and as such, can be used as a data for training and education. The overall score can also be used as an evaluation and comparison of results after interventions or between wards (or hospitals) and PIVC standards. Future electronic systems should thus allow for the *PIVC-miniQ* to become a part of patient safety-surveillance programs, which would allow for a continuous and automated PIVC quality surveillance system.

Some of the items required patient involvement (“Pain and tenderness” or “Where was the PIVC inserted”). The item “pain and tenderness” had the highest agreement between raters and showed that patients are consistent in their reporting independent of whom asks them. Such patient reported outcome measures that involve the patients can enable awareness and engagement in deviations in PIVC care and thereby break down a traditional professional culture [36]. Further, organization of today’s healthcare provides patient discharge from hospital with intravascular devices in place [41]. Empowering patients and family to be alert on the quality on such common devices improves patient safety. The *PIVC-miniQ* should thus next be tested for inter-rater variability between nurses and patients or nurses and relatives.

### Strengths and limitations

Previous literature has described that experienced raters are more coherent in their clinical judgements [42]. However, we consider our use of nurse educators; patients’ primary nurse or nurse students for the inter-rater testing as a strength. This heterogeneous group of nurses was chosen purposively to reflect the potentially users of the *PIVC-miniQ* in the future [43]. Our use of nursing students as raters could however have been a weakness due to potentially low experience and competence, which may have lowered the agreement. However, the subgroup of nursing students were too low to detect if there were any difference and nursing students have been used successfully in other PIVC assessment studies [17]. Nevertheless, we found it appropriate as neither nurses nor doctors receive any systematic education in evaluating PIVCs during school [44–46].

There were however differences between hospitals, and it seems like the former experience in the PIVC pre-study in Hospital A had positive effect on reliability. In the future, we therefore recommend some guidance of the raters before the *PIVC-miniQ* is used in daily practice.

Some items had low prevalence and low positive agreement. The low prevalence of each item is a factor known to produce lower positive agreement [47]. However, these items are extremely important for assessing the PIVC quality. Besides, the sum scores were most important in this study and these showed moderate to high agreement.

In our study some patient groups were excluded. Excluding isolated patients may have shortened the average time spent on each patient and thereby affected feasibility. In addition, due to the ethical constraints of the study we do not have information on patients not able to provide verbal consent, which may have led to a skewed prevalence of symptoms. However, when the patients are unable to participate (e.g. reduced consciousness), the *PIVC-miniQ* can still be used without self-report of “pain and tenderness” by calculating out of 15.

## Conclusion

Measuring patient safety must highlight validated tools with indicators that can be reported as rates [48]. This study finds the *PIVC-miniQ* to be a reliable and feasible audit tool measuring rates of PIVC problems. The *PIVC-miniQ* sum score allowed easy and efficient evaluations of effects with interventions to improve PIVC quality. Here, we provide a tool that can be used for surveillance of problems and measure continuous improvement in patient safety projects and thus ease evaluation of progress. Further testing in other countries are needed.

## Additional file

Additional file 1: **Table S1.** Overview of the development process of the *PIVC-miniQ.* **Figure S1.** *PIVC-miniQ.* **Figure S2**. Results from prevalence surveys using pre version *PIVC-miniQ. (DOCX 83 kb)*

## Data Availability

The datasets used during the current study are available from the corresponding author on reasonable request.

## Abbreviations

BSI: Bloodstream infection
CABSI: Catheter-associated bloodstream infections
EMS: Emergency Medical Services
G: Gauge
ICC: Intraclass correlation
ICU: Intensive Care Unit
IV: Intravenous
OMG-study: One Million Global Catheters study
PIVC: Peripheral intravenous catheter
*PIVC-miniQ*: Peripheral Intravenous Catheter mini Questionnaire
