# Correction to: Monitoring quality of care for peripheral intravenous catheters; feasibility and reliability of the peripheral intravenous catheters mini questionnaire (*PIVC-miniQ*)

**DOI:** 10.1186/s12913-020-05300-8

**Published:** 2020-05-14

**Authors:** Lise Husby Høvik, Kari Hanne Gjeilo, Stian Lydersen, Claire M. Rickard, Benedikte Røtvold, Jan Kristian Damås, Erik Solligård, Lise Tuset Gustad

**Affiliations:** 1 Department of Circulation and Medical Imaging, Norwegian University of Science and Technology (NTNU), Postbox 8905, 7491 Trondheim, Norway; 2 Gemini Center for Sepsis Research, St. Olavs Hospital and Norwegian University of Science and Technology (NTNU), Trondheim, Norway; 3 Clinic of Anaesthesia and Intensive Care, St. Olavs Hospital, Trondheim, Norway; 4 Department of Cardiothoracic Surgery, Department of Cardiology and National Competence Centre for Complex Symptom Disorders, St. Olavs Hospital, Trondheim University Hospital, Trondheim, Norway; 5 Department of Public Health and Nursing, Faculty of Medicine and Health Sciences, Norwegian University of Science and Technology (NTNU), Trondheim, Norway; 6 Regional Centre for Child and Youth Mental Health and Child Welfare, Department of Mental Health, Faculty of Medicine and Health Sciences, Norwegian University of Science and Technology (NTNU), Trondheim, Norway; 7 Alliance for Vascular Access teaching and Research, Menzies Health Institute Queensland, Griffith University, Brisbane, Australia; 8 School of Nursing and Midwifery, Griffith University, Brisbane, Australia; 9 Department of Anesthesia, Levanger Hospital, Clinic of Surgery, Nord-Trøndelag Hospital Trust, Levanger, Norway; 10 Centre of Molecular Inflammation Research, Department of Clinical and Molecular Medicine, NTNU, Trondheim, Norway; 11 Department of Infectious Diseases, St. Olavs Hospital, Trondheim, Norway; 12 Department of Medicine, Levanger Hospital, Clinic of Medicine and rehabilitation, Nord-Trøndelag Hospital Trust, Levanger, Norway

**Correction to: BMC Health Serv (2019) Res 19: 636.**

**https://doi.org/10.1186/s12913-019-4497-z**

Following publication of the original article [1], the authors identified an error in Table 3. In Table 3, column 8, the heading is “Positive agreement”. It should be “Absolute agreement” and the last column was wrong both in heading and in contents. The changes do not have impact on the interpretation of our data, discussion and conclusions.

The correct table is given below.

**Table 3:** Agreement and reliability results for the 16 items of the Peripheral Intravenous Catheters mini questionnaire (PIVC miniQ)

Item on the PIVC miniQ	neg	disagree	positive	sum	Negative agreement	Positive agreement	Absolute agreement	Scott’s pi
PIVC Pain and tenderness	168	15	22	205	0.957	0.746	0.927	0.703
Redness > 1 cm from insertion site	167	26	12	205	0.928	0.480	0.873	0.408
Swelling> 1 cm from insertion site	195	10	0	205	0.975	0.000	0.951	−0.025
Warmth at insertion site	199	5	1	205	0.988	0.286	0.976	0.273
Purulence	200	4	1	205	0.990	0.333	0.981	0.323
Streak/red line along the vein	199	4	2	205	0.990	0.500	0.981	0.490
Induration, hardness of tissue	197	7	1	205	0.983	0.222	0.966	0.205
Palpable hard vein beyond tip	190	13	2	205	0.967	0.235	0.937	0.202
Partial/complete dislodgement	201	2	2	205	0.995	0.667	0.990	0.662
Soiled with blood or fluids	146	21	38	205	0.933	0.784	0.898	0.716
Loose or lifting dressing edges	156	23	26	205	0.931	0.693	0.889	0.625
Fixed with tape only	202	3	0	205	0.993	0.000	0.985	−0.007
Blood in line	133	39	33	205	0.872	0.629	0.810	0.501
Insertion date not documented on PIVC dressing	85	18	102	205	0.904	0.919	0.912	0.823
Indication unknown	169	21	15	205	0.942	0.588	0.898	0.531
PIVC insertion date in is chart is lacking	132	36	37	205	0.880	0.673	0.824	0.553

*PIVC* Peripheral Intravenous Catheter
