# Supplementary material for: Monitoring quality of care for peripheral intravenous catheters; feasibility and reliability of the peripheral intravenous catheters mini questionnaire (PIVC-miniQ)
Source: BMC Health Serv Res. 2019 Sep 5;19:636. doi: 10.1186/s12913-019-4497-z (PMC6729030; doi:10.1186/s12913-019-4497-z)

Table A1 Overview of the development process of the PIVC miniQ

| Area of concern | The OMG study | Pre PiVC-miniQ | Ver 1 | Ver 2 | Ver 3 |
| --- | --- | --- | --- | --- | --- |
|  | Yes | No | Yes | Yes | Yes |
| Observations at Insertion site | 16 items:   1. No clinical symptoms 2. Pain tenderness on palpation 3. Redness > 1 cm from insertion site   4) Swelling > 1 cm from insertion site  5) Purulence  6) Itch/ rash under dressing  7) Blistering/ skin tears under dressing  8) Bruising/ dried blood around PIVC  9) Palpable hard vein cord beyond IV tip  10) Streak/ red line along vein  11) Induration/ hardness of tissues > 1 cm  12) Leaking PIVC,  13) Ekstravasation/ Infiltration  14) Blood in line  15) Partial dislodgement PIVC  16) Other | 14 items  Removed; OMG item 1 and 16.  Reason: we wanted to measure deviations from good PIVC quality and we wanted the questionnaire standardized (did not want the other option because this could lead to a lot of unwanted variation) | 8 items  Removed OMG item 15  **OMG items that were difficult to assess:**   - Removed OMG item 6 and 7   **Items difficult to differentiate:**   - Removed OMG Item 13 (often confused with item 4)   **Merged and moved**   - Item 8 and 12 merged with soiled bandage (area of concern: Bandage/ equipment) - Moved item 14 to bandage/ equipment | 9 items  OMG item 15 in again as we saw examples of this problem when we observed the insertion sites | 9 items  Unchanged from version 2 |
| Documentation of PIVC | 2 items:  Date and Time of PIVC insertion/ Not documented | 3 items | 2 items  Dropped time (hour) of insertion as this time rarely were documented | 2 items  Unchanged from ver 2 | 2 items  Unchanged from ver2 |
| Indication | 30 items  Reasons for PIVC insertion, different IV fluids today and IV medications today | 1 item;  “Indication unknown” | 1 item  Unchanged from pre *PIVC-miniQ* | 1 item  Unchanged from pre *PIVC-miniQ* | 1 item  Mark one of the following:  *“Indication unknown”*  **or**  *“Unclear medical condition, iv.fluids last 24 hours, epidural, x-ray or other procedures that require PIVC”*  The last item will not be added in the overall score. |
| Dressing/ equipment | 20 items  Dressing type, iv connectors, iv. administration set securement and dressing assessment | 3 items;  Moist and soiled with blood  Dry and soiled with blood  Loose or lifting edges | 6 items  Added (in addition to pre PIVC miniQ):  Non-sterile tape around PIVC  Blood in line  No date for PIVC insertion | Unchanged | Unchanged |
| Total items | 66 | 20 | 16 | 16 | 16 |
| Marking options | Check all that apply | Unchanged | Unchanged | Unchanged | Mark “yes” or “no” |
| Time recorded | No | No | Yes | Yes | Mark “yes” or “no” |

| **PIVC position/site:**  **Right Left** | **Catheter Gauge**  **PIVC size:** | **Where was the catheter inserted?**  *(ask patient if not documented)* |
| --- | --- | --- |
| □ Hand  □ Wrist  □ Forearm  □ Antecubital fossa  □ Foot  □ Head/neck  □ Upper arm | □ 24 G Yellow  □ 22 G Blue  □ 20 G Pink  □ 18 G Green  □ 16 G Grey  □ 14 G Brown/Orange | □ Ambulance/ ED  □ Emergency department  □ Operating room  □ General ward/ unit/ clinic/ ICU  □ Radiology/ Procedure room  □ Unknown |

| *PIVC-miniQ*  *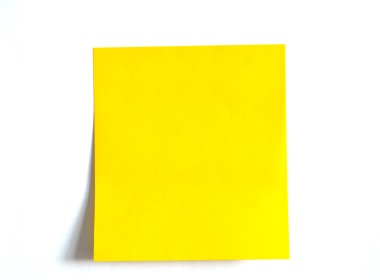*  Please obtain a separate survey for each PIVC.  Thank you! | \| Hospital/Site \|  \| \| --- \| --- \| \| Ward/Unit \|  \| \| Room/Bed Number \|  \| \| PIVC nr. \|  \| \| Age of patient \|  \| \| Gender of patient \| □ Male □ Female \| \| Date of PIVC insertion \|  \| \| Date of review \|  \| \| Rater-ID \|  \| |
| --- | --- | --- | --- | --- | --- | --- | --- | --- | --- | --- | --- | --- | --- | --- | --- | --- | --- | --- | --- |

| **PIVC site assessment:** | |  | **Iv dressing and iv connection assessment:** | | |
| --- | --- | --- | --- | --- | --- |
| Pain/tenderness on palpation  Redness > 1 cm from insertion site  Swelling > 1 cm from insertion site  Warmth  Purulence  Streak/ red line along vein  Induration/ hardness of tissues>1 cm   \| Palpabl Palpable hard vein beyond IV tip  elvis D Partial/ complete dislodgement PIVC \| \| --- \| | □ Yes  □ Yes  □ Yes  □ Yes  □ Yes  □ Yes  □ Yes  □ Yes  □ Yes | □ No  □ No  □ No  □ No  □ No  □ No  □ No  □ No  □ No | Soiled with blood or fluids  Loose or lifting edges  Tape only  Blood in line  PIVC insertion date not documented on dressing | □ Yes  □ Yes  □ Yes  □ Yes  □ Yes | □ No  □ No  □ No  □ No  □ No |

| **Indication:** | **Documentation:** |
| --- | --- |
| One of the following:  □ *Unclear medical condition, iv.fluids last 24 timer, epidural,*  *x-ray or other procedures that require PIVC.* *******  □ Indication unknown | Date of PIVC insertion  In patient chart is lacking □ Yes □ No |

Figure A1

***Not included in the sum-score**

Figure A2


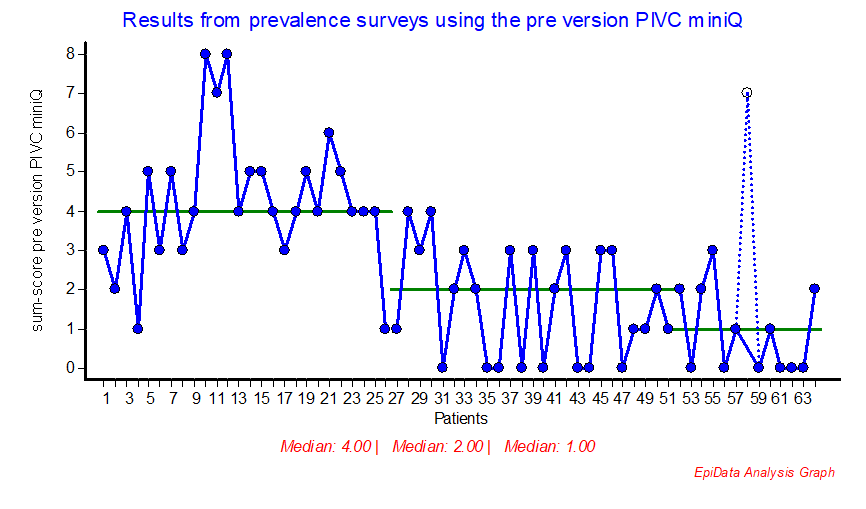

Supplement: Supplementary file 1 — Table S1. Overview of the development process of the PIVC-miniQ. Figure S1. PIVC-miniQ. Figure S2. Results from prevalence surveys using pre version PIVC-miniQ. (DOCX 83 kb) [file 12913_2019_4497_MOESM1_ESM.docx]
